# Axial F–Bi–O_v_ Electron Pump Drives Continuous Reconfiguration of Bi Sites for Efficient Photocatalytic N_2_ Reduction

**DOI:** 10.1002/advs.202520590

**Published:** 2026-03-28

**Authors:** Xiao Ge, Xinyi Wu, Hao‐tong Li, Xinya Liu, Jie‐jie Chen, Yuan Min, Xiaozhi Wang

**Affiliations:** ^1^ College of Environmental Science and Engineering Yangzhou University Yangzhou China; ^2^ Department of Environmental Science and Engineering University of Science and Technology of China Hefei China; ^3^ School of Inspection and Testing Certification Changzhou Vocational Institute of Engineering Changzhou Jiangsu China

**Keywords:** electronic structure, electron pumpoxygen vacancies, oxygen vacancies, photocatalytic N_2_ reduction, single‐atom catalysts

## Abstract

The electronic structure precision of single‐atom catalysts (SACs) represents a decisive factor limiting advancements in photocatalytic nitrogen reduction (NRR) efficiency. This study addresses this issue by simultaneously introducing an axial fluoride ligand (F^−^) and engineering surface oxygen vacancies (O_v_) around atomically dispersed Bi centers supported on W_18_O_49_ (denoted as FBWO). The axial fluoride ligand withdraws electron density away from the Bi site, increasing surface hydrophobicity and forming a surface dipole. This dipole lowers the conduction band while promoting side‐on N_2_ chemisorption. Meanwhile, photo‐induced O_v_ accumulate electrons around Bi site, forming a continuous F–Bi–O_v_ “electron pump” that reduces the activation energy for the first proton‐electron transfer from 1.69 eV (on pristine W_18_O_49_ with single Bi sites, BWO) to 0.87 eV (on FBWO). These synergistic electronic and energetic modifications enable the material to achieve a visible‐light NH_3_ production rate of 354.2 *µ*mol g^−1^·h^−1^–8.4 times that of pristine W_18_O_49_ and twice that of BWO—surpassing all recently reported Bi‐ and W‐based photocatalysts for N_2_ reduction. This work provides a unified design strategy that integrates ligand‐field engineering with defect chemistry, facilitating the targeted development of SACs into high‐performance photocatalysts for sustainable ammonia production.″

## Graphical abstract

Axial Fluorine and Light‐Induced Oxygen Vacancies Synergize as an Electron Pump to Lower N_2_ Activation Energy and Enable Sustained NH_3_ Synthesis on FBWO.

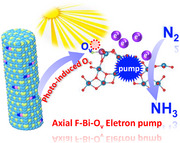

## 1 Introduction

Ammonia (NH_3_) is indispensable for global food security as a key component of agricultural fertilizers and is increasingly recognized as a carbon‐neutral energy carrier [1]. However, its conventional synthesis via the energy‐intensive Haber‐Bosch process—operating under extreme conditions (15–25 MPa, 673–873 K), consuming approximately 1–2% of global energy and accounts for 1.8% of annual CO_2_ emissions [1, 2, 3]. These interlinked energy, climate, and food‐system challenges underscore the urgent need for sustainable alternatives capable of operating under mild conditions.

NRR, which utilizes sunlight, water, and atmospheric N_2_ at ambient pressure, presents an attractive solution. However, its practical deployment faces two critical hurdles [4, 5]. First, the high bond dissociation energy of the N≡N triple bond (941 kJ·mol^−1^) necessitates a substantial overpotential, while the competing hydrogen evolution reaction (HER) occurs at nearly identical redox potentials, severely reducing Faradaic efficiency [6]. Second, the low aqueous solubility of N_2_ (6.5 × 10^−4^ mol·L^−1^ at 298 K) requires catalysts to rapidly anchor and activate the molecule before it diffuses away. Despite advances, state‐of‐the‐art photocatalysts—including oxides, chalcogenides, metal‐organic frameworks (MOFs), and graphitic carbon nitride—achieve NH_3_ yields rarely exceeding 100–300 µmol g^−1^·h^−1^, far below industrial requirements [7, 8, 9, 10]. Therefore, this requires considerable effort to develop highly effective catalysts that can meet the strict performance standards needed for practical NRR production [11].

The fundamental obstacle lies in the absence of well‐defined active sites capable of simultaneously polarising the N_2_ molecule and stabilising the *N–NH intermediate [5, 12, 13]. SACs, which are created by anchoring isolated metal atoms onto support, offer a solution by optimizing atomic utilization efficiency and establishing uniformly distributed active sites [14]. In SACs configurations, every metal atom is directly accessible to reactants, which is a significant advantage over nanoparticles [15, 16]. In nanoparticles, a large proportion of interior atoms are inaccessible due to aggregation. The unique structural framework of SACs redefines catalytic paradigms. It fully exposes the coordination environment of each metal center to the reaction environment, thereby enhancing both reactivity and selectivity [17, 18]. This enables precise tuning of *d*‐band centers [5, 19, 20], spin states, and local electric fields [5, 21, 22, 23, 24, 25]. Our previous research demonstrates that even minor ligand‐field modifications—induced by heteroatom donors or neighboring vacancies—can reduce the activation barrier for the N_2_→N_2_H transition by over 0.5 eV, effectively distinguishing between “active” and “inactive” catalysts [5]. Experimentally, Ru_1_/TiO_2_, Fe_1_‐N_4_/C and Cu_1_ sites have validated this principle, but their NH_3_ production rates remain below 200 *µ*mol·g^−1^·h^−1^ due to poorly defined or thermally unstable ligand environments [26, 27, 28].

Bismuth (Bi), a non‐toxic and Earth‐abundant p‐block element, has recently attracted attention for NRR owing to its unique electronic structure [29]. Specifically, Bi's 6p orbitals possess the unique ability to back‐donate electron density into the π* orbitals of the N_2_ molecule [30]. This characteristic sets Bi apart from transition metals, which often suffer from strong *H (hydrogen adsorbate) adsorption issues that inadvertently promote the competing hydrogen evolution reaction (HER) [29, 31]. Unlike transition metals that tend to bind hydrogen strongly and thus favor HER over NRR, Bi's 6p orbitals selectively interact with the π antibonding orbitals of N_2_. This selective interaction not only facilitates the preferential activation of N_2_ but also effectively suppresses the HER pathway. However, isolated Bi atoms on oxide supports suffer from weak N_2_ adsorption and prohibitively high activation barriers, resulting in negligible NH_3_ under visible light. Therefore, it is crucial to have a support that can stabilize Bi atoms and also facilitate a continuous supply of electrons [32, 33]. Non‐stoichiometric W_18_O_49_ is an ideal candidate, as its intrinsic O_v_ localize electrons, suppress carrier recombination, and enhance charge transfer to adsorbates [34, 35].

In this study, we introduce a transformative dual‐strategy that simultaneously introduces an axial fluoride ligand and photo‐induced O_v_ around single Bi atoms supported on W_18_O_49_. This approach constructs an unprecedented axial F–Bi–O_v_ “electron pump” mechanism. First, the electronegative F ligand withdraws electron density from Bi, generating a surface dipole that enhances N_2_ chemisorption. Second, photoelectrons accumulate at O_v_ sites for continuous delivery to adsorbed N_2_. This synergistic modulation uniquely reduces the *N_2_→N_2_H activation barrier, achieving an excellent NH_3_ production rate of 354.2 *µ*mol·g·cat^−1^·h^−1^. Unlike previous single‐modification methods, this integrated ligand‐defect engineering establishes a universal principle for converting inert supports into highly active SACs platforms, representing a conceptual advancement beyond conventional active‐site design.

## 2 Results and Discussion

### 2.1 Materials Synthesis and Characterization

The synthesis of these samples is achieved through a solvothermal method, where F atoms serve as precursors to modulate the electronic structures of the Bi active centers, as schematically represented in Figure S1. The X‐ray diffraction (XRD) (Figure S2) peaks for all samples are accurately indexed to the monoclinic W_18_O_49_ (JCPDS No. 71–2450) with a crystal cell depicted in Figure S3, suggesting the presence of a single Bi site supported by W_18_O_49_. Furthermore, Figure S4a,b reveals a transmission electron microscope (TEM) image showcasing a sphere with a 500 nm diameter, composed of numerous nanorods. Additionally, high‐resolution TEM (HRTEM) images exhibit ordered lattice fringes with a spacing of 0.38 nm, attributable to the (010) planes, signifying that FBWO grows along the (010) direction of W_18_O_49_ (Figure S4c). The FBWO nanostructures adopt a flower‐like morphology composed of nanorods that affords a higher surface area (Figure S5) and, consequently, exposes a high density of accessible Bi single‐atom sites, thereby potentially enhancing catalytic performance.

**FIGURE 1:**
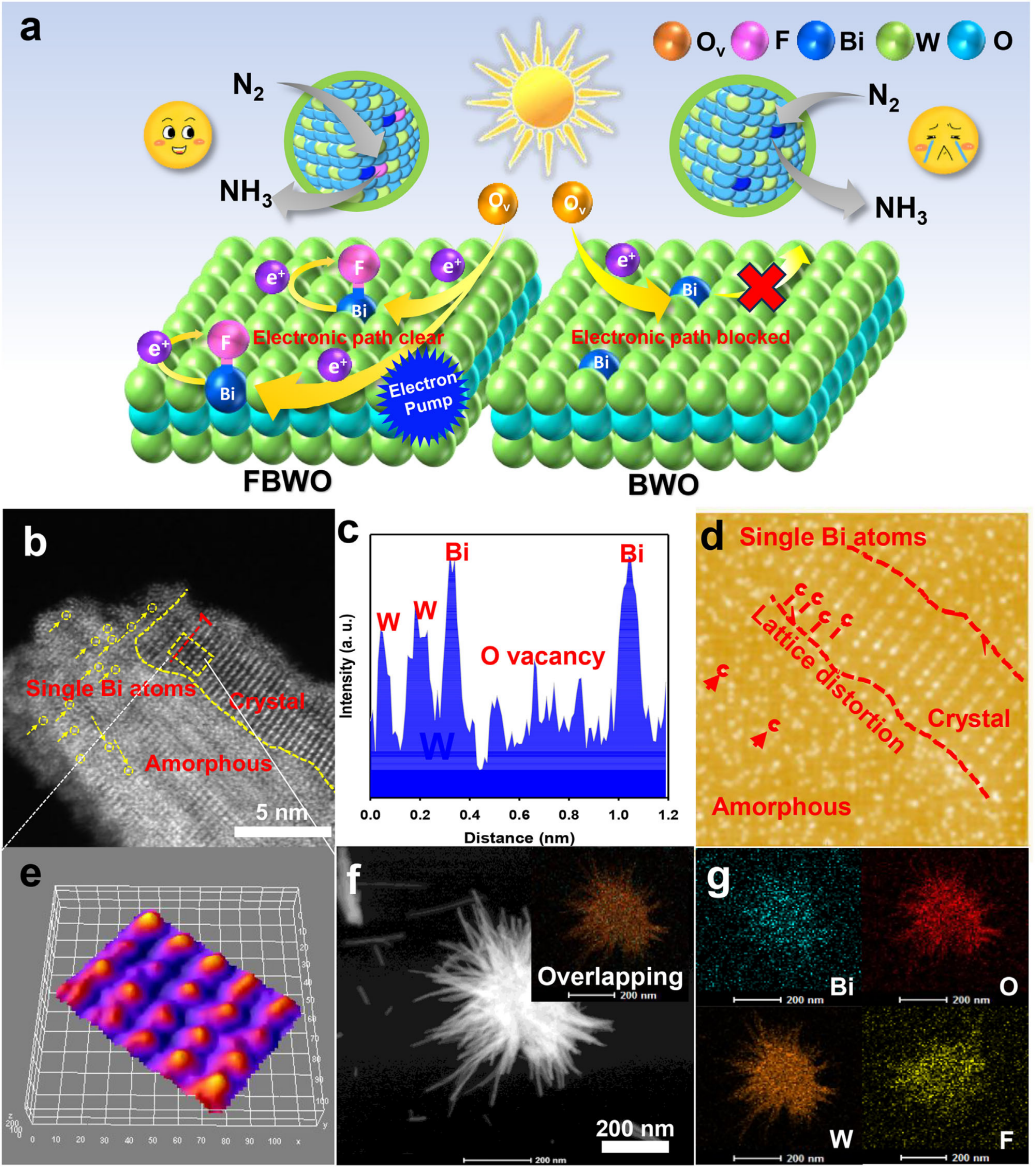
Catalyst illustration and characterization. (a) Axial F–Bi–O_v_ “Electron Pump” for Photocatalytic N_2_ Reduction on FBWO. (b) AC‐HAADF‐STEM image of FBWO. (c) The corresponding intensity of line 1 profile of dotted line in (b). (d) The simulated image of (b). (e) 3D intensity surface plot of the marked areas in (b)1. (f, g) TEM image of FBWO and corresponding EDS elemental mappings.

To gain a deeper understanding of the arrangement of Bi atoms, aberration‐corrected high‐angle dark‐field scanning TEM (AC‐HAADF‐STEM) was conducted, along with geometric phase analysis and image simulation analysis of FBWO (Figure 1b–d). The AC‐HAADF‐STEM imagery of FBWO revealed that individual Bi sites (highlighted by yellow circles) are dispersed in a directional manner on W_18_O_49_ nanoparticles, as evident in Figure 1b. The corresponding lines in Figure 1b indicate that Bi single atoms are loaded onto the surface of W_18_O_49_, as illustrated in Figure 1c. Additionally, as shown in Figure 1d, FBWO experienced a transition from crystalline to amorphous with lattice distortion, which would cause a large number of OVs on W_18_O_49_ after Bi and F incorporation. Furthermore, Figure 1e shows the spatial distribution of Bi (represented by large and bright atoms) and W (represented by small and dark atoms) atoms within the FBWO. To verify the precise elemental composition of FBWO, energy dispersive X‐ray spectroscopy (EDS) mapping was performed, as presented in Figure 1f,g. The distribution of Bi, O, W, and F elements, corresponding to the scanning TEM (STEM) image, is consistent with the uniform single Bi sites observed, aligning with the comprehensive XPS spectrum of FBWO (Figure S6).

**FIGURE 2:**
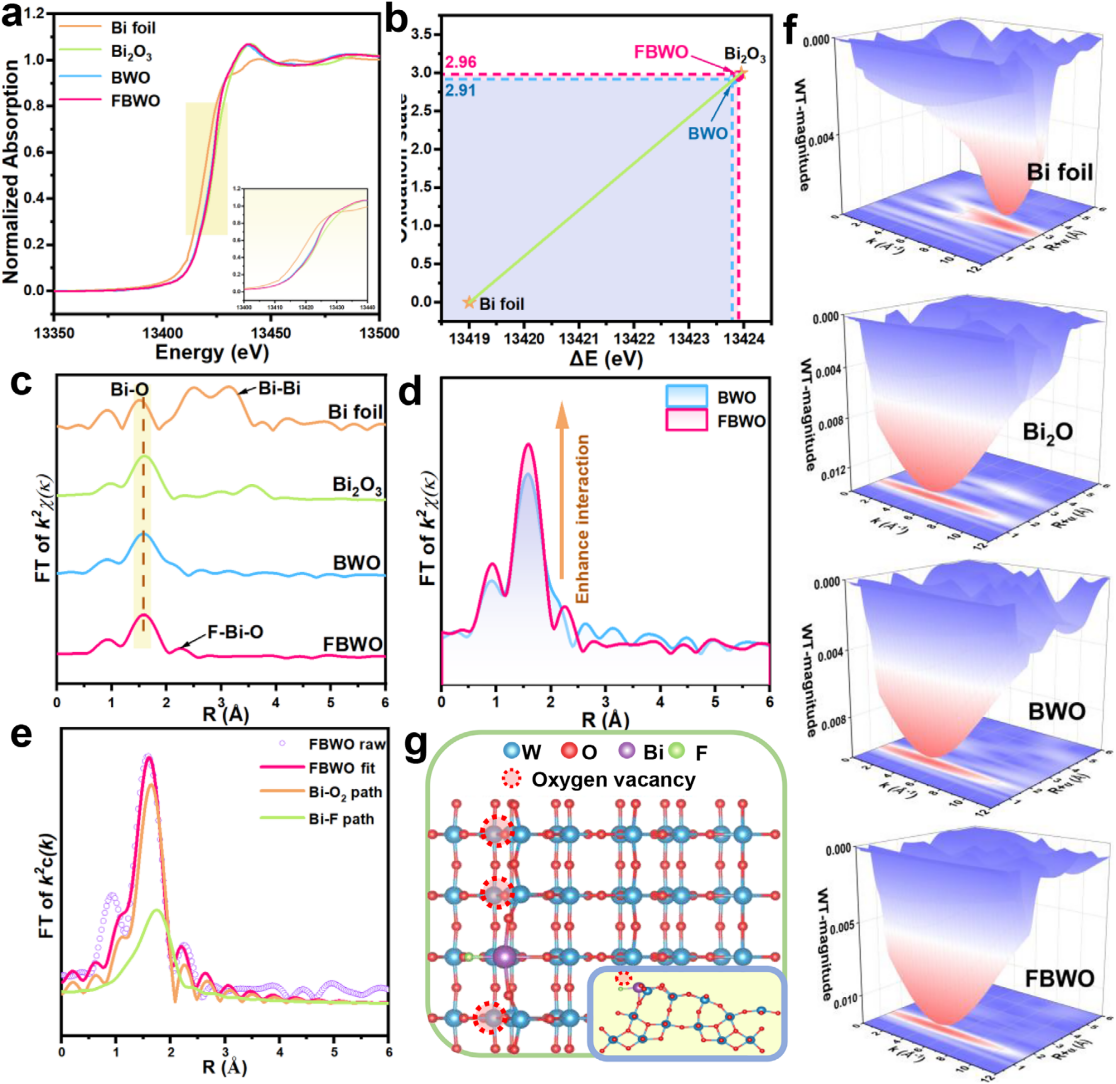
Electronic Structure and Coordination Environment Analysis. (a) XANES spectra of the normalized Bi L_3_‐edge of Bi foil, Bi_2_O_3_, BWO and FBWO. (b) The calculated Bi oxidation states from the XANES spectra of FBWO and BWO along with those of Bi foil and Bi_2_O_3_ as references. (c) EXAFS fitting curves of Bi foil, Bi_2_O_3_, BWO and FBWO in the K‐space. (d) Comparison of the Bi L_3_‐edge of BWO and FBWO. (e) EXAFS fitting curves of the Bi L_3_‐edge of FBWO. (f) Wavelet transform (WT) plots of Bi foil, Bi_2_O_3_, BWO and FBWO. (g) The structural illustration of FBWO.

X‐ray absorption near‐edge structure (XANES) and X‐ray photoelectron spectroscopy (XPS) were employed to investigate the chemical states and coordination environments of Bi, O, F, and W in FBWO. As shown in Figure 2a, the XANES spectra reveal that the absorption edges of BWO and FBWO lie between those of Bi foil and Bi_2_O_3_, indicating that the valence state of Bi in these materials is intermediate between Bi^0^ and Bi^3+^. Notably, the introduction of axial F induces a significant shift of the absorption edge toward higher energy in FBWO compared to BWO, as highlighted in the inset of Figure 2a. This shift is attributed to the formation of Bi─F bonds at the Bi sites, which elevates the valence state of Bi atoms in FBWO. Additionally, the F 1s spectrum (Figure S7) corroborates the presence of Bi─F dangling bonds. Quantitative analysis of the oxidation states of Bi in BWO and FBWO yielded values of +2.91 and +2.96, respectively (Figure 2b), aligning with the above conclusions and validating the existence of Bi─F bonds at the Bi sites. Finally, the fitting results for W_18_O_49_, BWO, and FBWO (Figure S8) demonstrate that the introduction of Bi does not alter the valence state of W.

Extended X‐ray absorption fine structure (EXAFS) analysis and XPS spectra provided deeper insights into the local coordination environment of Bi in FBWO. The Fourier transform (FT) of the EXAFS spectra (Figure 2c; Figures S9–S11 and Table S1) reveals a prominent peak at 1.52 Å in FBWO, corresponding to Bi‐O/F coordination. Compared to BWO, the Bi─O/F bonds in FBWO exhibit stronger interactions, attributed to the formation of Bi─F dangling bonds (Figure 2d). These findings, combined with the absence of Bi‐W or Bi‐Bi peaks in both BWO and FBWO, confirm the atomic dispersion of single Bi sites in these materials.

Further analysis of the FT‐EXAFS spectrum of BWO (Figure 2e; Figures S10, S11, and Table S1) and the fitted parameters (Table S1) indicates that isolated Bi atoms in BWO are coordinated with two O atoms. In contrast, FBWO exhibits coordination of three O atoms and one F atom per Bi atom (Figure 2e; Table S1). The positive shift observed in the Bi 4f spectra after F introduction (Figure S9) is consistent with the formation of axial F─Bi bonds. Wavelet transform (WT) analysis of EXAFS, which offers high sensitivity in both k and R ranges, further corroborates these findings. As depicted in Figure 2f, the WT of EXAFS for BWO and FBWO shows maximum intensity at ≈5.0 Å^−1^, distinct from that of Bi foil and Bi_2_O_3_. Therefore, these results highlight the critical role of Bi─F dangling bonds in enhancing the interaction between active centers and supports in FBWO. Based on the overall analysis of the FBWO's coordination environment described above, the detailed structure of the material is presented in Figure 2g.

**FIGURE 3:**
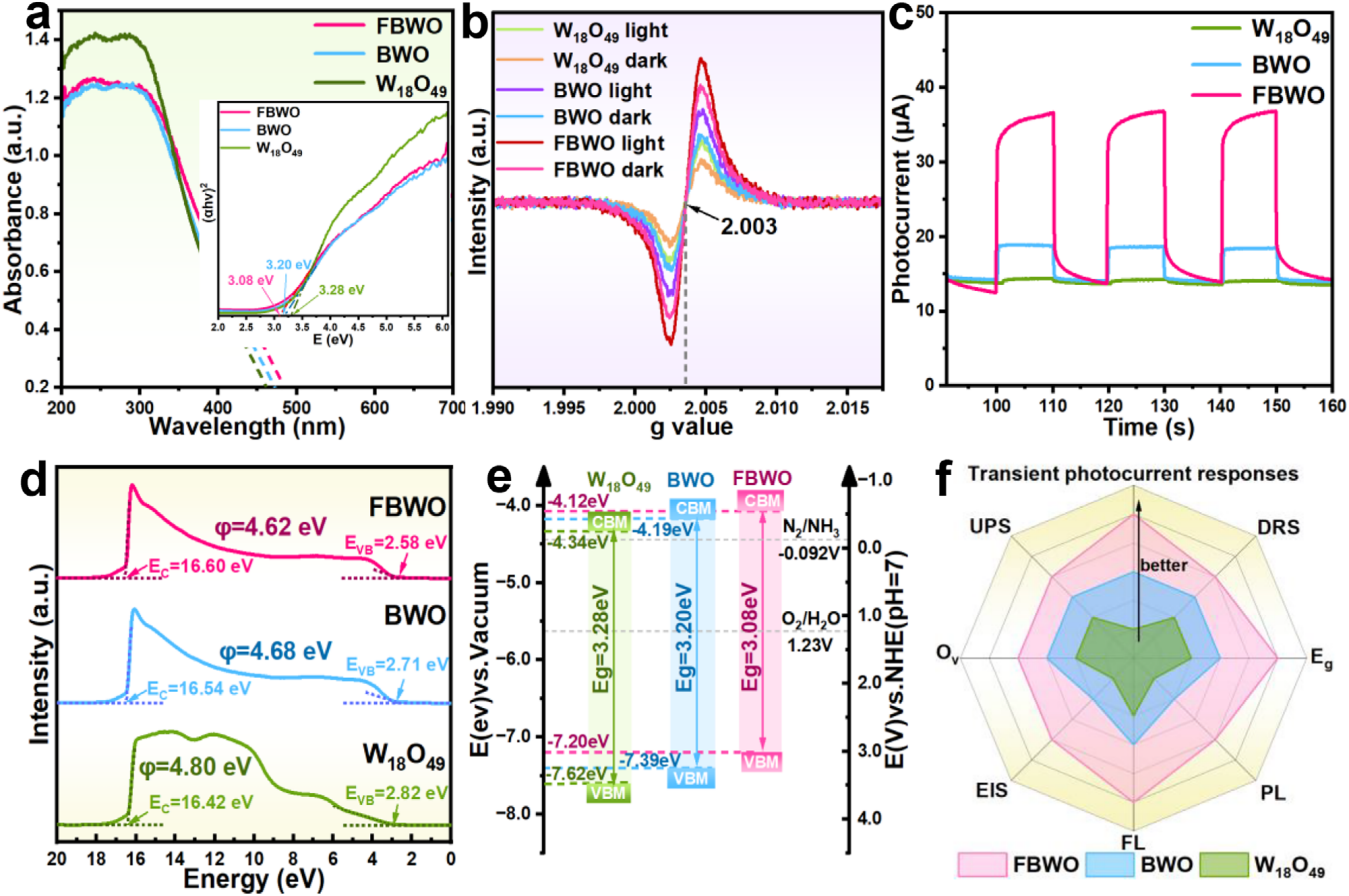
Optical Properties of the resulting samples. (a) UV–vis absorption spectra of the as‐prepared W_18_O_49_, BWO and FBWO; insets: corresponding Tauc plots. (b) Low‐temperature EPR spectra of the as‐prepared W_18_O_49_, BWO and FBWO before and after light irradiation. (c) Transient photocurrent responses of the three types of catalysts under 1064 nm light irradiation. (d) UPS spectra. (e) Electronic energy‐level diagram of the as‐prepared W_18_O_49_, BWO and FBWO. (f) Comparisons of optical characterization of the resulting samples.

The diffuse reflectance spectroscopy (DRS) UV‐vis absorption spectra of W_18_O_49_, BWO, and FBWO (Figure 3a) reveal distinct optical property modifications. While pristine W_18_O_49_ exhibits visible light absorption, the absorption edges of BWO and FBWO undergo a pronounced redshift. By extrapolating the Tauc plots derived from the DRS data (Figure 3a inset), the bandgap energies (*E*_g_) of W_18_O_49_, BWO, and FBWO are determined to be 3.28, 3.20, and 3.08 eV, respectively. The band‐gap narrowing in FBWO originates from the synergistic interplay of O_v_ insertion and F‐induced electronic‐structure modulation.

In addition, to investigate the surface O_v_ dynamics, low‐temperature electron paramagnetic resonance (EPR) analysis was conducted (Figure 3b). Pristine W_18_O_49_ exhibits a characteristic O_v_ signal at g = 2.003, which intensifies under light irradiation—indicative of photoinduced O_v_ generation. Following Bi incorporation in BWO, the O_v_ signal is markedly intensified, indicating that extensive oxygen depletion from the lattice substantially elevates the O_v_ concentration. Notably, FBWO demonstrates the most pronounced O_v_ signal, both in the dark and under illumination, correlating with the positive shift in O 1s XPS spectra (Figure S12). This O_v_ enrichment suppresses charge recombination by providing trapping sites for photogenerated carriers.

Transient photocurrent measurements (Figure 3c) corroborate these findings, with FBWO exhibiting the highest photocurrent response. This remarkable boost in charge‐separation stems from the concerted action of photo‐generated O_v_ and F‐tuned Bi electronic states, which jointly accelerate interfacial charge transfer. The gradual photocurrent rise/decay for FBWO (Figure 3c) reflects dynamic accumulation/release of photoelectrons in O_v_ traps, while BWO's sharp response indicates transient charge separation without storage. This temporal behavior corroborates the F–Bi–O_v_ electron pump mechanism. Complementary evidence from photoluminescence (PL) spectra (Figure S13) shows a weakened band‐to‐band emission peak at 490 nm for FBWO, confirming reduced radiative recombination. Furthermore, electrochemical impedance spectroscopy (EIS; Figure S14) reveals a smaller Nyquist semicircle diameter for FBWO, indicating diminished charge transfer resistance and improved conductivity.

The band‐edge alignment derived from ultraviolet photoelectron spectroscopy (UPS; Figure 3d,e) provides critical insight into the photocatalytic mechanism. The work functions (Φ) of W_18_O_49_, BWO, and FBWO decrease progressively from 4.80 to 4.62 eV, accompanied by a downward shift in valence band (VB) positions (2.82 eV→2.58 eV). Combined with the reduced bandgap, these changes elevate the conduction band (CB) edge of FBWO, positioning it above the N_2_/NH_3_ redox potential.

The comparative analysis of comprehensive optoelectronic performance metrics for the resulting samples is systematically presented in Figure 3f. Notably, the FBWO composite demonstrates superior optoelectronic characteristics across multiple evaluation criteria, including enhanced photocurrent generation, optimized impedance characteristics, photo‐generated O_v_, prolonged fluorescence lifetime (FL) decay dynamics and reduced work function values compared to other formulations. Therefore, this strategic band structure optimization endows FBWO with enhanced photogenerated electron reducing power, thereby amplifying its catalytic activity for nitrogen reduction.

### 2.2 Photocatalytic Nitrogen Reduction Performance

**FIGURE 4:**
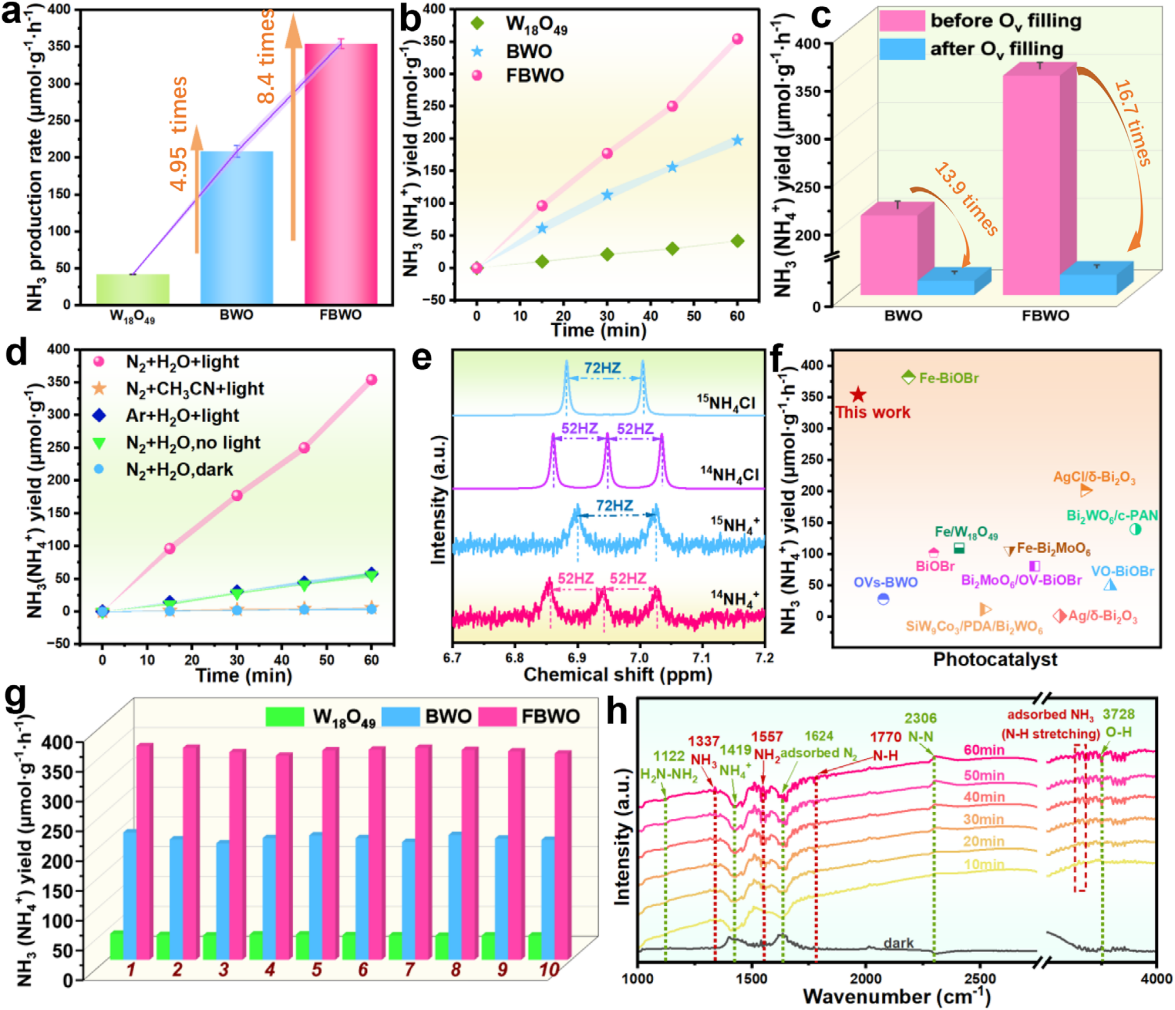
Photocatalytic N_2_ Fixation Performance. (a) Photocatalytic ammonia production rates by W_18_O_49_, BWO and FBWO in 1 h. Light *source*: 300 W Xe lamp with a cut‐off, λ > 400 nm; Reaction solution: 100 mL of deionized water; Catalyst: 10 mg. (b) Quantitative determination of NH_3_ generated by the as‐prepared W_18_O_49_, BWO and FBWO under visible light (λ > 400 nm) irradiation. (c) NH_3_ generated by the as‐prepared BWO and FBWO before and after O_v_ filling. (d) NH_3_ generated by the as‐prepared FBWO in different contrast conditions. (e) 1H NMR (500 MHz) spectra of FBWO fed by ^14^N_2_, ^15^N_2_ with full‐spectrum irradiation, and 1H NMR (500 MHz) spectra of ^14^NH_4_Cl and ^15^NH_4_Cl. (f) NH_3_ generated by the as‐prepared FBWO in different contrast catalysts. (g) Photocatalytic ammonia production rates in the first 10 h for cyclic tests of W_18_O_49_, BWO and FBWO. (h) In situ DRIFTS spectra from as‐prepared FBWO during the photocatalytic N_2_ fixation.

To investigate the modulation of the electronic structure of single Bi sites by asymmetric fluorine coordination and O_v_ in FBWO, we evaluated its photocatalytic performance in nitrogen fixation. The experiments were conducted under full‐spectrum irradiation in N_2_‐saturated water without sacrificial agents (Figure S15). As shown in Figure 4a, FBWO exhibits exceptional photocatalytic activity, achieving an ammonia yield of 354.21 *µ*mol·g·cat^−1^·h^−1^. This is 8.4 times higher than pristine W_18_O_49_ (42.12 *µ*mol·g·cat^−1^·h^−1^) and nearly double that of the single Bi site catalyst (197.44 *µ*mol·g·cat^−1^·h^−1^). Time‐dependent measurements (Figure 4b) further confirm that ammonia production correlates directly with irradiation time, while immediate cessation of ammonia formation upon light shutdown (Figure S16) underscores the photocatalytic nature of the process. Notably, no N_2_H_4_ or H_2_ byproducts were detected, highlighting the high selectivity for NH_3_ production.

Annealing FBWO and BWO in air selectively eliminates O_v_ and yields a pronounced and parallel decline in catalytic activity. As shown in Figure 4c and Figure S17, the ammonia production rate falls by a factor of 13.9 for BWO and 16.7 for FBWO, evidencing that O_v_ serve as the primary source of localized electrons that modulate the electronic structure of the Bi sites and sustain the proton–electron transfers required for photocatalytic NH_3_ formation. Additionally, replacing H_2_O with aprotic CH_3_CN (Figure 4d) abolishes N_2_ fixation, indicating that protons for NH_3_ formation originate from water. Similarly, substituting N_2_ with Ar reduces the fixation rate, confirming that NH_3_ stems from N_2_ rather than adventitious nitrogen. Isotopic labeling with ^15^N_2_ and ^1^HNMR analysis (Figure 4e) further corroborate this conclusion, with the characteristic doublet (J_N−H_ = 72 Hz) confirming ^15^NH_4_^+^ formation [36].

Importantly, by comparing the photocatalytic ammonia production performance under similar reaction conditions, FBWO achieves NH_3_ production rates that are among the highest reported for Bi‐ and W‐based photocatalysts, surpassing most literature values obtained under similar visible‐light, ambient‐pressure conditions (Figure 4f; Table S2) [37, 38, 39, 40, 41, 42, 43, 44, 45, 46]. Meanwhile, its stability was also evaluated over 10 h of continuous operation (Figure 4g), with no decline in ammonia yield, demonstrating excellent reusability and durability. Meanwhile, the stability of Bi single‐atom sites in FBWO was also confirmed through XPS analysis before and after 10 cycles of photocatalytic reactions. The Bi 4f, F 1s, and O 1s spectra show negligible changes, indicating that the Bi‐F coordination and overall electronic environment are preserved throughout the reaction cycles (Figure S18). These results demonstrate the structural robustness of the catalyst, ensuring that Bi remains atomically dispersed and active. Furthermore, the apparent quantum efficiency (QE) of FBWO is spectrally synchronized with its absorption envelope across all examined wavelengths, culminating in a striking peak of 4.31% at 365 nm (Figure S19). This wavelength marks both the optimum for photocatalytic performance and positions FBWO as a pre‐eminent candidate for NH_3_ photo‐synthesis. Notably, F incorporation significantly enhanced the FBWO's surface hydrophobicity, as evidenced by the contact angle increasing from 13.2° to 19.1° (Figure S20). This observation corroborates that the high electronegativity and low surface energy of F atoms synergistically reduce competitive water adsorption, thereby improving N_2_ adsorption efficiency through surface property modulation [47].

To elucidate the N_2_ hydrogenation mechanism on FBWO, in situ diffuse reflectance infrared fourier transform spectroscopy (DRIFTS) was employed. Under UV‐vis irradiation, time‐dependent DRIFTS spectra (Figure 4h) reveal progressive signal intensification. Within 60 min, absorption bands emerge in the 1000–4000 cm^−1^ range. The band at 3728 cm^−1^ corresponds to O‐H stretching, while the 1770 cm^−1^ peak reflects N‐H bending modes [48, 49]. NH_4_^+^ and NH_3_ are identified at 1419 and 1337 cm^−1^ [50, 51, 52], respectively. A prominent peak at 2306 cm^−1^ indicates N≡N, which is absent in pristine N_2_ FTIR spectra [48, 49]. This signal arises from the disruption of N≡N triple bonds via O_v_‐N≡N interactions, suggesting that light‐induced O_v_ progressively weaken N≡N bonds until cleavage into NH_3_/NH_4_^+^ [53]. The band at 1122 cm^−1^ attributed to H_2_N‐NH_2_ implies a symmetric alternating pathway for nitrogen fixation on FBWO [5, 14, 51].

Based on DRIFTS analyses, a detailed reaction pathway for nitrogen fixation on FBWO has been established. The process commences with selective adsorption of N_2_ molecules at isolated Bi active sites, which are characterized by axial coordination of F and enhanced density via surface defect engineering. This structural configuration facilitates N≡N bond elongation and polarization, effectively priming the inert triple bond for catalytic activation. The adsorbed N_2_ species then undergoes sequential hydrogenation through a series of proton‐coupled electron transfer reactions, progressing through intermediate states *NH and *NH_2_ before yielding gaseous NH_3_. Notably, transient formation of NH_4_^+^, detected as distinct spectroscopic features, provides direct evidence for the stepwise proton‐coupled electron transfer mechanism. The overall kinetics of the nitrogen‐reduction reaction are governed by the electronic architecture of single‐atom Bi sites. This architecture is precisely tuned through axial fluoride ligation and further modulated by photo‐induced oxygen‐vacancy engineering. The resulting orbital alignment between the Bi centers and nitrogen‐containing intermediates markedly improves interfacial charge‐transfer efficiency. Each hydrogenation step is thereby accelerated, which lowers the energy barrier of the entire nitrogen‐reduction pathway. Therefore, the combined effects of axial F coordination, defect‐mediated site distribution, and single‐atom electronic modulation establish a low‐energy channel for electrocatalytic nitrogen fixation.

### 2.3 DFT Calculations

**FIGURE 5:**
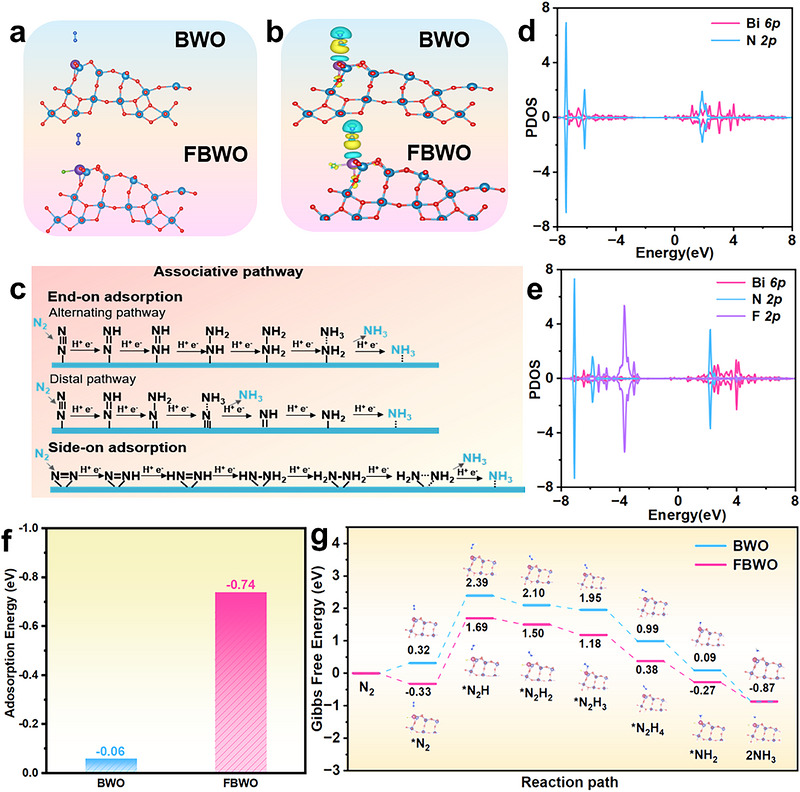
DFT insights into N_2_ adsorption and activation on BWO and FBWO. (a) N_2_ adsorption configurations on BWO and FBWO models; (b) The charge density difference between BWO and FBWO; (c) Three proposed pathways of reduction of N_2_ to NH_3_ on the catalyst surface; (d) Projected density of states (PDOS) of the Bi 6*p* orbitals in BWO together with the N 2*p* orbitals of adsorbed N_2_; (e) PDOS of Bi 6*p* and F 2*p* orbitals in FBWO together with the N 2*p* orbitals of adsorbed N_2_; (f) Adsorption energies of N_2_ on the catalyst surface; (g) Gibbs free energy profiles for reduction of N_2_ to NH_3_ on the surface of BWO and FBWO.

Atomic‐level DFT calculations provide a coherent explanation for the enhanced N_2_‐reduction activity of FBWO compared with BWO, tracing the process from adsorption geometry to the Gibbs free‐energy landscape. N_2_ preferentially migrates toward the Bi center and adopts an end‐on configuration (Figure 5a). Although end‐on is the global minimum, a metastable side‐on configuration bridging Bi and a neighboring oxygen vacancy is also accessible (Figure 5c), suggesting that both modes may participate under catalytic conditions.

The ordering of hydrogenation steps is modulated by adsorption geometry and local electronic environment. Operando infrared spectroscopy identifies a dominant associative route, wherein proton–electron pairs are delivered alternately to opposite nitrogen atoms (*N_2_H→*NH‐NH→*NH_2_‐NH_2_). Consistent with this assignment, the Gibbs free‐energy profiles (Figure 5g) show that the potential‐determining step (*N_2_ →*NNH) requires 1.69 eV on BWO but only 0.87 eV on FBWO, demonstrating that fluorination substantially lowers the thermodynamic barrier for N_2_ activation. Once *N_2_H is formed, subsequent hydrogenation steps proceed energetically downhill on FBWO, whereas they remain comparatively uphill and sluggish on BWO. In addition, side‐on adsorption with a symmetric charge distribution across both nitrogen atoms may enable a concerted sequence (*N_2_→*HN = NH →*H_2_N–NH_2_) followed by simultaneous N–N cleavage, although this pathway is less competitive thermodynamically.

To rationalize these energetic trends, charge density difference maps (Figure 5b) reveal the origin of the stronger binding on FBWO. The F ligand withdraws electron density from Bi atoms, creating a pronounced Bi–F–O_v_ dipole. This polarization depletes charge around Bi (blue) and enriches it around F (yellow), which in turn polarizes the bound N_2_. The effect is quantified by the adsorption energies (Figure 5f): –0.74 eV on FBWO compared with only –0.06 eV on BWO, confirming markedly stronger chemisorption. Complementarily, projected density of states analysis (Figure 5d,e) shows that the introduction of F 2*p* states leads to stronger hybridization between Bi 6*p* and N 2*p* orbitals, with additional overlap from F 2*p*. This hybridization produces a more localized and reactive electronic environment around the Bi–F–O_v_ motif, thereby enhancing electron donation into the N_2_ π* orbital and promoting its activation. Overall, F‐driven polarization and *p*‐state mixing at the Bi–F–O_v_ site coherently rationalize the stronger N_2_ binding on FBWO and prime the adsorbate for the initial hydrogenation step.

Thus, the calculations reveal that fluorination transforms BWO from an inert substrate into an active catalyst through a stepwise electronic mechanism. This charge redistribution localizes electrons at the Bi‐F‐O_v_ motif and enhances their reactivity toward N_2_ activation. Simultaneously, photo‐induced O_v_ accumulate excess electrons adjacent to the Bi center. Acting in concert, the axial F ligand, Bi sites, and neighboring O_v_ sites form an axial F–Bi–O_v_ “electron pump” that continuously channels photoelectrons into the π* orbital of end‐on bound N_2_, thereby enabling efficient nitrogen fixation.

## 3 Conclusions

In conclusion, we demonstrate that electronic structure modulation at single‐atom Bi sites can be realized by simultaneously installing an axial F ligand and photo‐generated O_v_ within W_18_O_49_. The axial F withdraws electron density from Bi sites, while the adjacent vacancy accumulates a localized electron reservoir. These two effects synergistically form an axial F–Bi–O_v_ electron pump that continuously supplies reducing equivalents to the π* orbital of end‐on‐bound N_2_. This mechanism significantly reduces the activation energy barrier for *N_2_→*N_2_H conversion, thereby accelerating the overall nitrogen fixation process. By transforming an initially inert substrate into a highly efficient photocatalyst, this ligand‐defect strategy provides a universal approach for designing atomically precise photocatalytic systems capable of sustainable ammonia production under mild conditions.

## Conflicts of Interest

The authors declare no conflicts of interest.

## Supporting information




**Supporting File**: advs75047‐sup‐0001‐SuppMat.pdf.

## Data Availability

The data that support the findings of this study are available on request from the corresponding author. The data are not publicly available due to privacy or ethical restrictions.
