# Supplementary material for: Axial F–Bi–Ov Electron Pump Drives Continuous Reconfiguration of Bi Sites for Efficient Photocatalytic N2 Reduction
Source: Adv Sci (Weinh). 2026 Mar 28;13(33):e20590. doi: 10.1002/advs.202520590 (PMC13271607; doi:10.1002/advs.202520590)
Supplement: Supplementary file 1 — Supporting File: advs75047‐sup‐0001‐SuppMat.pdf. [file ADVS-13-e20590-s001.docx]

**Supporting Information**

**Axial Fluorination-Oxygen Vacancy Synergy Drives Continuous Electron Pumping for Efficient Photocatalytic N_2_ Reduction**

Xiao Ge^a+^*, Xinyi Wu^a+^, Hao-tong Li^b+^, Xinya Liu^c^, Jie-jie Chen^b^, Yuan Min ^b*^, Xiaozhi Wang^ac*^

[a] College of Environmental Science and Engineering, Yangzhou University, Yangzhou 225000, China;

[b] Department of Environmental Science and Engineering, University of Science and Technology of China, Hefei 230026, China.

[c] School of Inspection and Testing Certification, Changzhou Vocational Institute of Engineering, Changzhou, Jiangsu, China.

[+] These authors contributed equally to this work.

*E-mail: [gexiao@yzu.edu.cn](mailto:gexiao@yzu.edu.cn); [minyuan@ustc.edu.cn](mailto:minyuan@ustc.edu.cn); [xzwang@yzu.edu.cn](mailto:xzwang@yzu.edu.cn)

**1. Materials:**

Tungsten hexachloride (WCl_6_), Ethanol (CH_3_CH_2_OH), Bismuth trichloride (BiCl_3_), Sodium fluoride (NaCl), Hydrochloric acid (HCl), Ammonium sulfate [(NH_4_)_2_SO_4_], Sodium nitrosoferricyanide (Na_3_Fe(CN)_6_·H_2_O), Salicylic acid (C_7_H_6_O_3_), Sodium citrate (Na_3_C_6_H_5_O_7_), Sodium hydroxide (NaOH), Sodium hypochlorite (NaClO), Ammonium chloride (NH_4_Cl), Deuterodimethyl sulfoxide (C_2_H_6_OS, DMSO), acetonitrile (C_2_H_3_N) were all of analytic grade and sourced from Sinopharm Chemical Reagent Co. Ltd. (Shanghai, China). Deionized water, with a resistivity exceeding 18.2 MΩ·cm, was produced using a JL-RO100 Millipore-Q Plus water purifier and utilized consistently in our experiments. None of the chemicals underwent any additional purification prior to use.

**2. Characterizations:**

Transmission electron microscope (TEM) and high-resolution TEM (HR-TEM) images were captured using a Hitachi-7700 microscope operated at 100 kV. To visualize the isolated single Bi atoms, an aberration-corrected HAADF-STEM (specifically, a FEI Tecnai G2 F20) was employed at an operating voltage of 200 kV. XRD patterns were recorded on a Rigaku Miniflex-600 X-ray powder diffractometer, utilizing Cu Kα radiation. XPS measurements were performed on a PHI 5000 Verasa X-ray photoelectron spectrometer from ULAC-PHI, Inc., with Al Kα serving as the excitation source.

For ICP-OES analysis, a Varian ICPS-720 1 (with a detection limit of 1 ppm) was used exclusively to determine the Bi content in BWO and FBWO. Prior to testing, 50 mg of each sample was dissolved in a mixture of concentrated nitric acid (HNO_3_; 6 mL), perhydrol (H_2_O_2_; 6 mL), and hydrofluoric acid (HF; 0.5 mL) in a 50 mL polytetrafluoroethylene container. The container was then sealed in a stainless steel autoclave and heated to 190 °C for 10 hours to ensure complete dissolution. The resulting solutions were diluted with ultrapure water in a 25 mL volumetric flask for analysis. This method was employed solely for Bi quantification and was not used for nitrogen content determination.

EPR measurements were conducted using a Bruker A300 spectrometer, operating at a modulation frequency of 100 kHz. Prior to testing, the samples were dispersed in a solution, and spin-trapping agents, namely DMPO and TEMP, were introduced for prompt analysis. As for the Bi K-edge XAFS analyses, these were carried out utilizing Si (111) crystal monochromators at the BL14W Beamline of the Shanghai Synchrotron Radiation Facility (SSRF) in Shanghai, China. Before beamline analysis, the samples were securely placed in aluminum holders and sealed with Kapton tape film.

The XAFS spectra were then recorded at room temperature, employing a 4-channel Silicon Drift Detector (SDD) Bruker 5040. Specifically, the Bi K-edge extended X-ray absorption fine structure (EXAFS) spectra were captured in transmission/fluorescence mode. Notably, minimal variations were observed in the line-shape and peak position of the Bi K-edge XANES spectra between two consecutive scans of the same sample. Data was gathered in transmission mode for Bi foil and Bi_2_O_3_, while fluorescence excitation mode was used for BWO and FBWO. All spectra were recorded under ambient conditions. The XAFS spectra of these reference samples were documented in transmission mode and subsequently processed and analyzed using the Athena software suite.

**3. Experimental Section**

**(1) Synthesis of W_18_O_49_**

200 mg of WCl_6_ was dissolved in 60 ml of C_2_H_5_OH, followed by ultrasonic treatment for 10 minutes to produce a clear light yellow solution. This solution was then transferred to the lining of a polytetrafluoroethylene high-pressure reactor, sealed tightly, and reacted at 200 ℃ for 12 hours. Once the reaction was complete and the mixture had cooled to room temperature, it was centrifuged to separate the precipitate, which was then washed several times with ethanol. Finally, the powder sample of W_18_O_49_ was obtained after vacuum drying.

**(2) Synthesis of BWO**

To prepare BWO samples, we used BiCl_3_ as the Bi precursor. The mixed solution of Bi was prepared by adding 20 mg of BiCl_3_ and 0.5 ml of HCl to 9 ml of C_2_H_5_OH. The synthesis process for BWO follows the same steps as that for W_18_O_49_, with one additional step: before transferring the light yellow solution to the polytetrafluoroethylene lining, we added the mixed solution of Bi with varying concentrations. The powder sample of BWO was then obtained by following the remaining steps in the synthesis of W_18_O_49_.

**(3) Synthesis of FBWO**

To prepare FBWO samples, we used BiCl_3_ as the Bi precursor and NaF as the F precursor. The mixed solution of Bi and F was prepared by adding 20 mg of BiCl_3_, 0.5 ml of HCl, and 10 mg of NaF to 9 ml of C_2_H_5_OH. The synthesis process for FBWO follows the same steps as that for W_18_O_49_, with one additional step: before transferring the light yellow solution to the polytetrafluoroethylene lining, we added the mixed solution of Bi and F with varying concentrations. The powder sample of FBWO was then obtained by following the remaining steps in the synthesis of W_18_O_49_.

**(4) Synthesis of W_18_O_49_ and FBWO without** **the oxygen vacancy**

To saturate the coordination metal and investigate the importance of oxygen vacancy, we encapsulated the oxygen vacancy within the material. Specifically, we weighed 200 mg of W_18_O_49_ or FBWO and positioned it in the center of a porcelain boat. The material was then annealed in an air atmosphere at 400 ℃ for 1 hour. Once it cooled to room temperature, we obtained a powder sample enriched with oxygen vacancy.

**(5) Photocatalytic N_2_ reduction**

The photocatalytic nitrogen reduction reaction was conducted in a quartz reaction tank under a nitrogen atmosphere. A xenon lamp source, with a power density of 2000 mW·cm^-2^, served as a simulated solar light source. The temperature of the entire reaction system was maintained at 25 ℃ using a circulating water cooling system. 10 mg of catalyst was added to 100 ml of pure water, and the mixture was treated with ultrasound for 2 minutes to ensure uniform dispersion of the catalyst in the water, forming a suspension. This suspension was then stirred under dark conditions while bubbling high-purity nitrogen into the reaction system at a rate of 200 ml/min for 30 minutes to achieve a nitrogen-saturated solution. 1 ml of the reaction solution was collected using a syringe. Following this, the xenon lamp light source was activated to simulate solar irradiation, and high-purity nitrogen was continuously bubbled into the system at a reduced rate of 100 ml/min. 1 ml of the reaction solution was withdrawn using a syringe every 15 minutes. After centrifugation, the supernatant was obtained and diluted 20 times. The concentration of NH_4_^+^ in the solution was determined using the salicylic acid method. Additionally, 1 M H_2_SO_4_ and 1 M NaOH were utilized to adjust the pH of the reaction solution, allowing for an investigation into the effects of pH on N_2_ reduction efficiency.

Nessler’s reagent spectrophotometry was employed to quantify NH_3_ evolution and thus evaluate UV–vis-driven N_2_-fixation performance; concurrently, ion-chromatography was used to cross-validate the standard curve. All catalytic activity data were collected from three independent experiments, with error bars in figures representing standard deviations.

**(6) Cyclic Experiment Methodology**

The stability of the photocatalyst was evaluated through consecutive recycling experiments for NH_3_ production. In each cycle, 10 mg of FBWO catalyst was dispersed in 100 mL of deionized water within a 150 mL quartz reactor. The suspension was ultrasonicated for 5 minutes to ensure uniform catalyst dispersion, followed by purging with high-purity N_2_ gas (200 mL·min⁻^1^) for 30 minutes in the dark to achieve a N_2_-saturated solution and remove residual atmospheric gases.

The photocatalytic reaction was then initiated by irradiating the mixture with a 300 W xenon lamp (λ > 400 nm, 2000 mW·cm⁻^2^) under continuous N_2_ bubbling (150 mL·min⁻^1^). The system temperature was maintained at 25 ± 1 °C using a circulating water cooling apparatus. After 1 hour of irradiation, the reaction was terminated and the catalyst was recovered by vacuum filtration, washed three times with deionized water and twice with ethanol to remove any adsorbed species, and dried in a vacuum oven at 60 °C for 6 hours. The regenerated catalyst was then redispersed in fresh deionized water (100 mL) for the subsequent cycle under identical conditions. NH_3_ concentration in the filtrate was quantified using Nessler’s reagent spectrophotometry after each cycle.

**4. The ^15^N_2_ isotope labeling experiment**

A 60 ml quartz reactor was prepared by adding 10 mg of catalyst and 40 ml of pure water, followed by ultrasonic treatment for 2 minutes to achieve a homogenous suspension. The air within the reactor was then evacuated using a vacuum pump, and high-purity argon was introduced. This process of evacuating and refilling with argon was repeated twice to ensure a pure environment. Afterward, high-purity ^15^N_2_ was injected into the reactor. Immediately following this, a xenon lamp light source was activated to mimic solar irradiation for a duration of 2 hours. Upon completion of the irradiation, the reaction solution was promptly centrifuged to separate and collect the supernatant. The pH of this supernatant was adjusted to 2 using hydrochloric acid. For 1H NMR analysis, 0.1 ml of dimethyl sulfoxide-D6 was added to 0.5 ml of the pH-adjusted solution.

**5. Computational Methods**

Density functional theory (DFT) calculations were carried out using the Vienna Ab initio Simulation Package (VASP)^1^. Structural optimizations were performed within the generalized gradient approximation (GGA) using the Perdew–Burke–Ernzerhof (PBE) functional^2^. Spin polarization was considered in all computational analyses. The core region’s wave function was described through the projector augmented wave (PAW) method^3^. The weak Van der Waals interactions were described by dispersion-corrected PBE+D3 approach^4^. The Brillouin zone was sampled using a 1×1×1 Monkhorst–Pack k-point mesh, which is sufficient for the large supercell; denser meshes were tested to confirm convergence. Structural relaxations were carried out until the total energy converged to 10^-5^ eV/atom and the forces on each atom were smaller than 0.02 eV/Å. The adsorption energy (${\text{∆}\text{E}}_{\text{Adsorption}}$) was determined by the following equation:

$$\text{∆E}_{\text{Adsorption}}\text{ = }E_{M^{*}}\text{- }\text{E}_{\text{M}}\text{ - }E_{*}$$

where $E_{M^{*}}$,$\text{E}_{\text{M}}$, and $E_{*}$ denote the total energies of the adsorbate–substrate system, the clean substrate, and the energy of the bare surface, respectively.


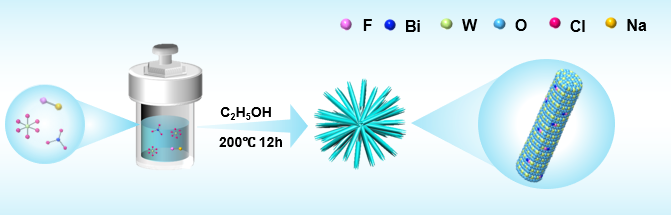


**Figure S1.** Schematic representation of FBWO synthesis.


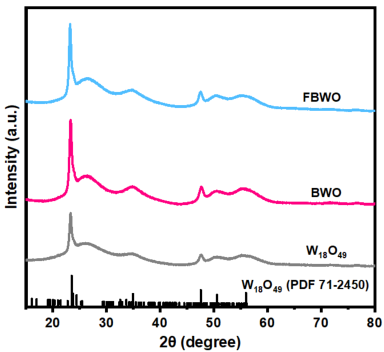


**Figure S2.** XRD patterns of the resulting samples.

**Figure S3.** The structural illustration of FBWO.

**Figure S4.** (a-b) TEM images of FBWO at different magnifications. (c) HRTEM images of FBWO.


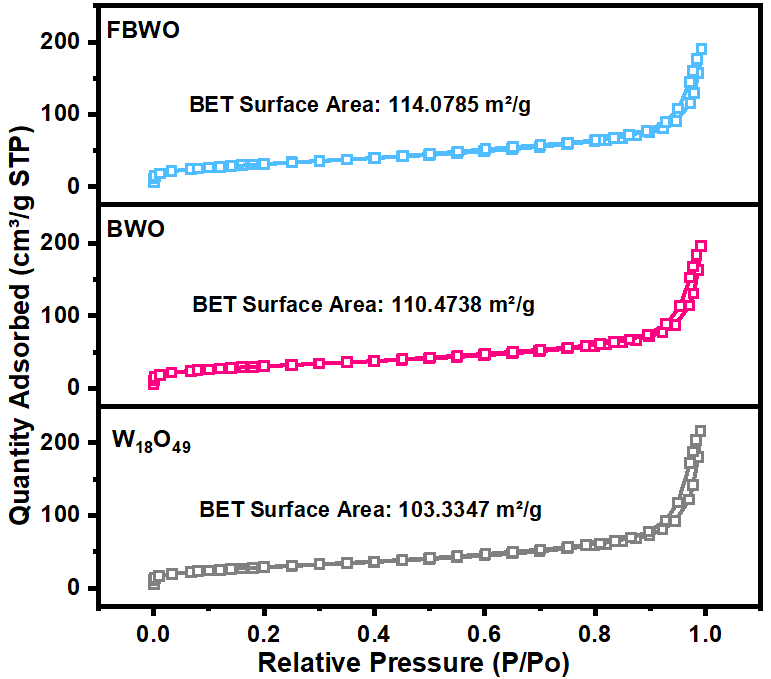


**Figure S5.** N_2_ adsorption–desorption isotherms of W_18_O_49_, BWO and FBWO.


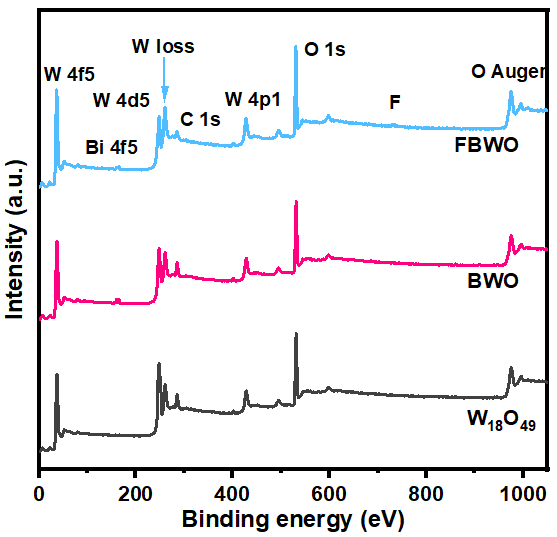


**Figure S6.** Survey XPS spectra of W_18_O_49_, BWO and FBWO.


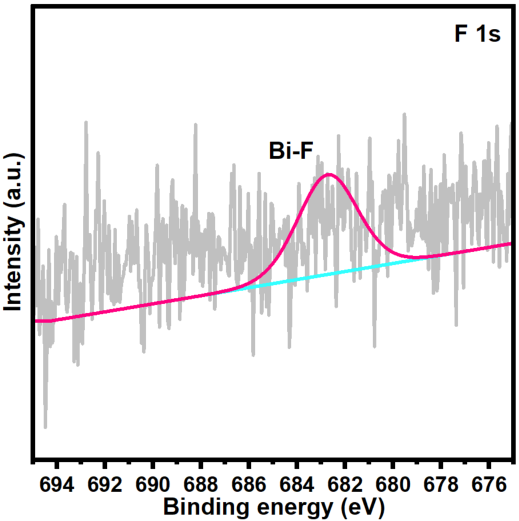


**Figure S7.** F1s XPS spectrums of FBWO.


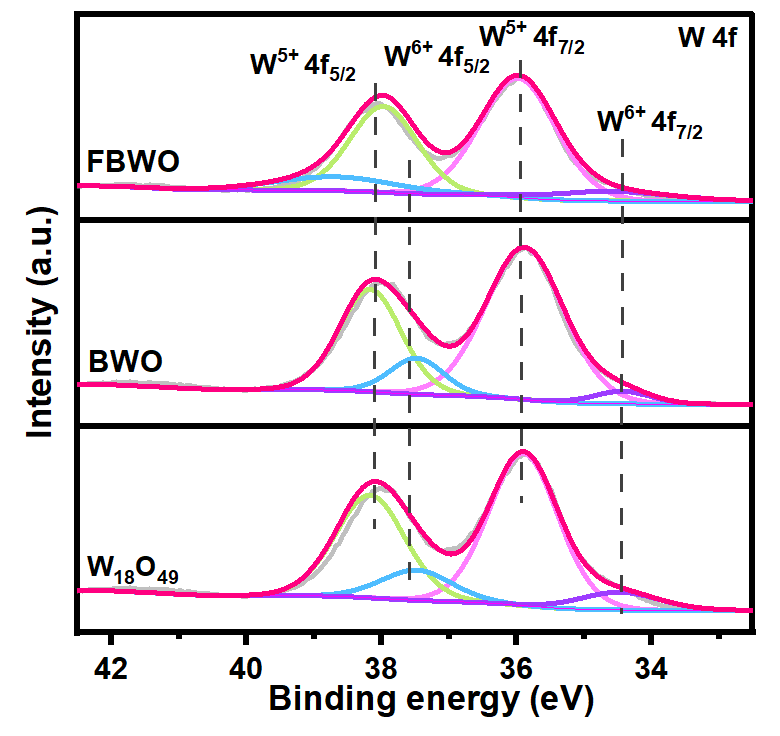


**Figure S8.** W 4f XPS spectra of W_18_O_49_, BWO and FBWO.


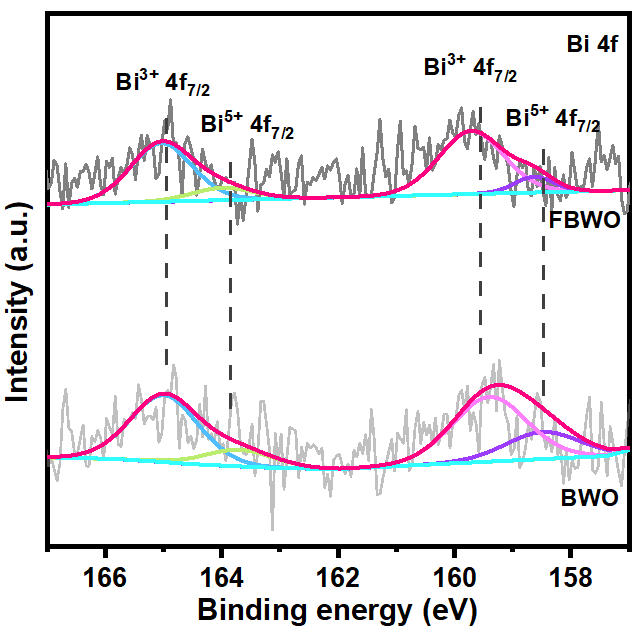


**Figure S9.** Bi 4f XPS spectra of BWO and FBWO.

**Figure S10.** Corresponding EXAFS fitting curves for BWO.

**Figure S11.** Corresponding EXAFS fitting curves for FBWO.


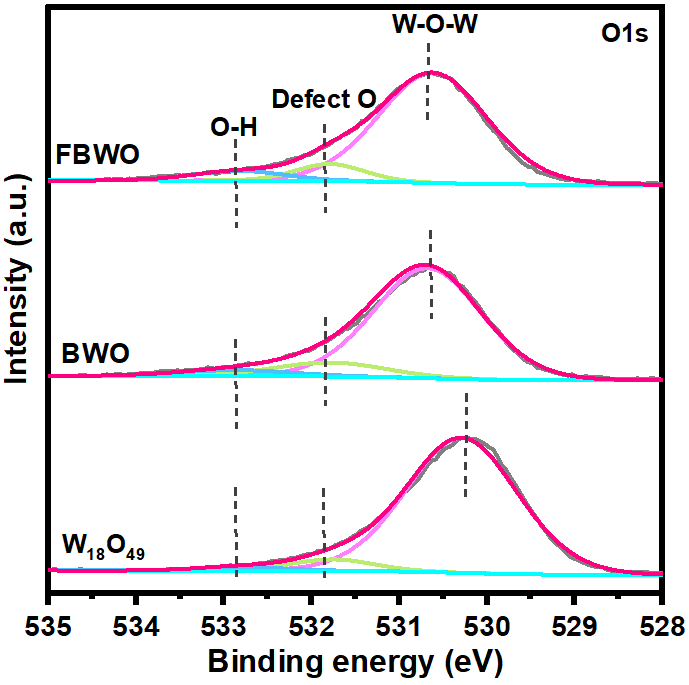


**Figure S12.** O 1s XPS spectrums of W_18_O_49_, BWO and FBWO.


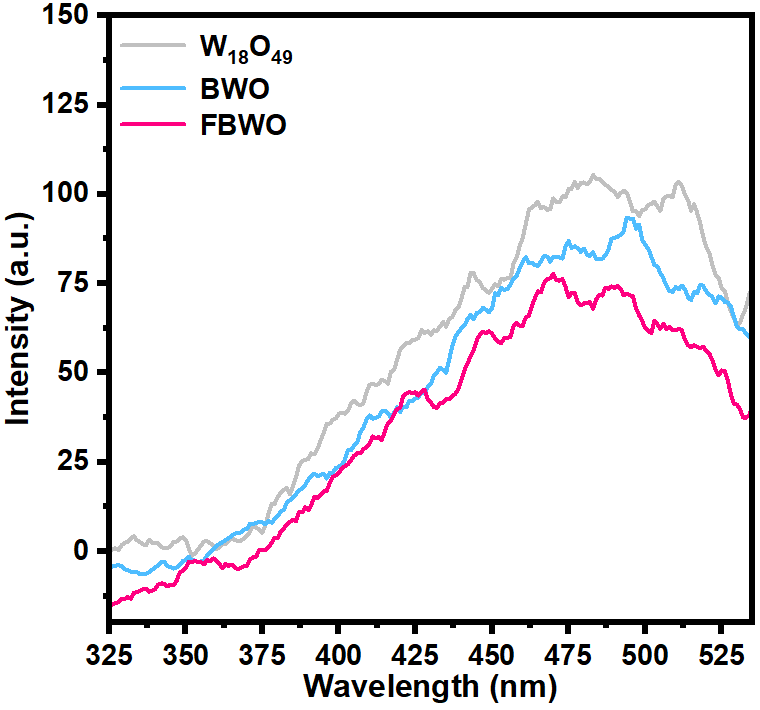


**Figure S13.** Room temperature steady-state photoluminescence spectra of W_18_O_49_, BWO and FBWO.


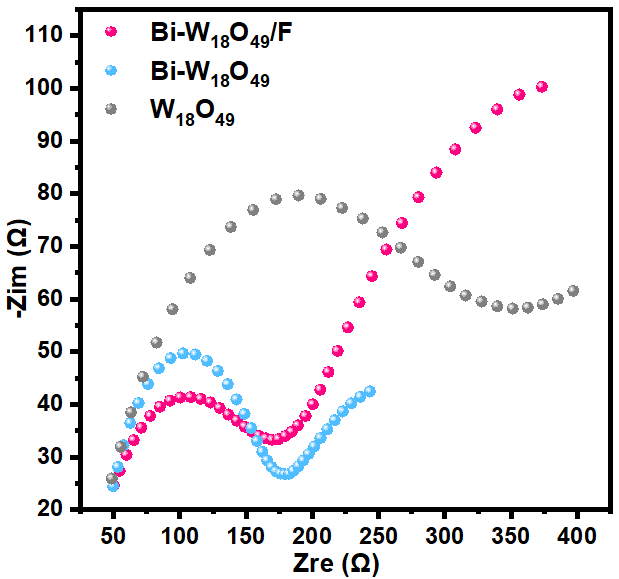


**Figure S14.** Impedance curves of W_18_O_49_, BWO and FBWO under visible light irradiation.

**Figure S15.** Calibration curves for NH_4_^+^ determined with Nessler’s reagent and by ion chromatography. Nessler’s reagent spectrophotometry was employed to quantify NH_3_ evolution and thus evaluate UV–vis-driven N_2_-fixation performance; concurrently, ion-chromatography was used to cross-validate the standard curve.


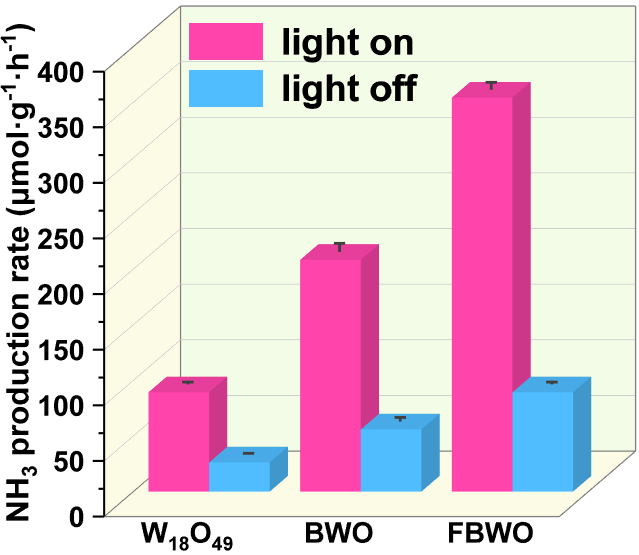


**Figure S16.** NH_3_ generated by the as-prepared W_18_O_49_, BWO and FBWO before and after light irradiation.


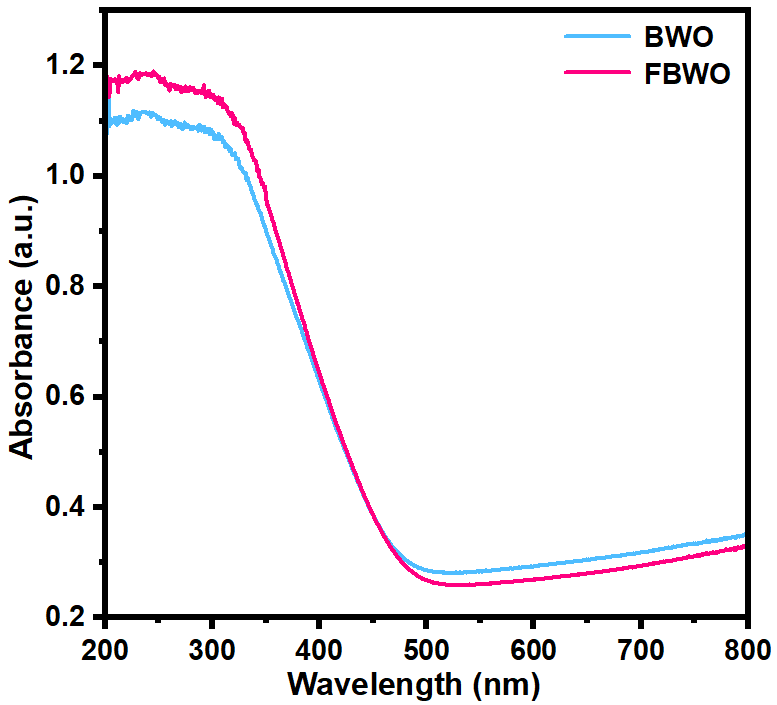


**Figure S17.** UV–vis absorption spectra of BWO and FBWO after oxygen-vacancy filling.

**Figure S18.** XPS of FBWO before and after 10 cycles of photocatalytic reactions.


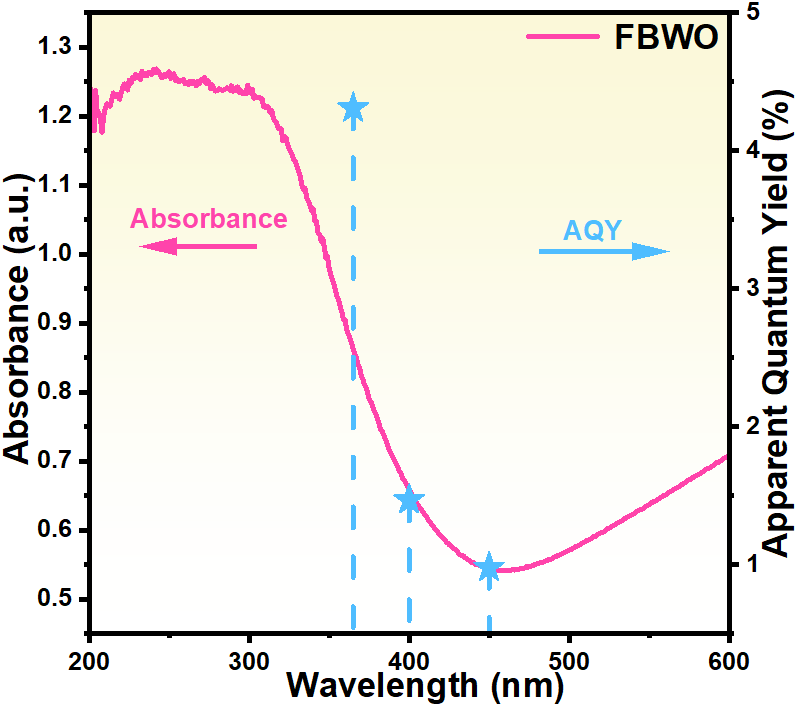


**Figure S19.** Q_E_ of FBWO under monochromatic light with corresponding optical absorption spectra.

**Figure S20.** The contact angle measurement of water on W_18_O_49_, BWO and FBWO.


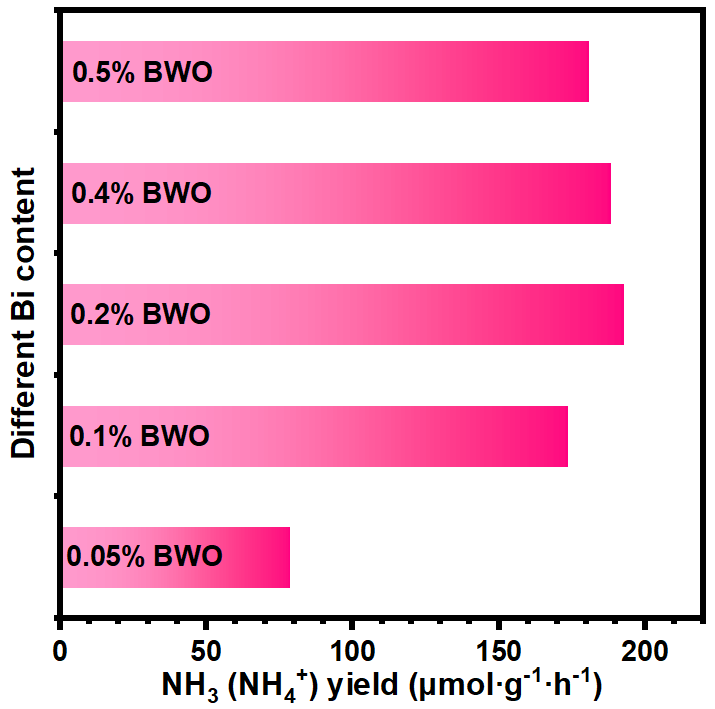


**Figure S21.** NH_3_ evolution over FBWO photocatalysts with varying Bi content.


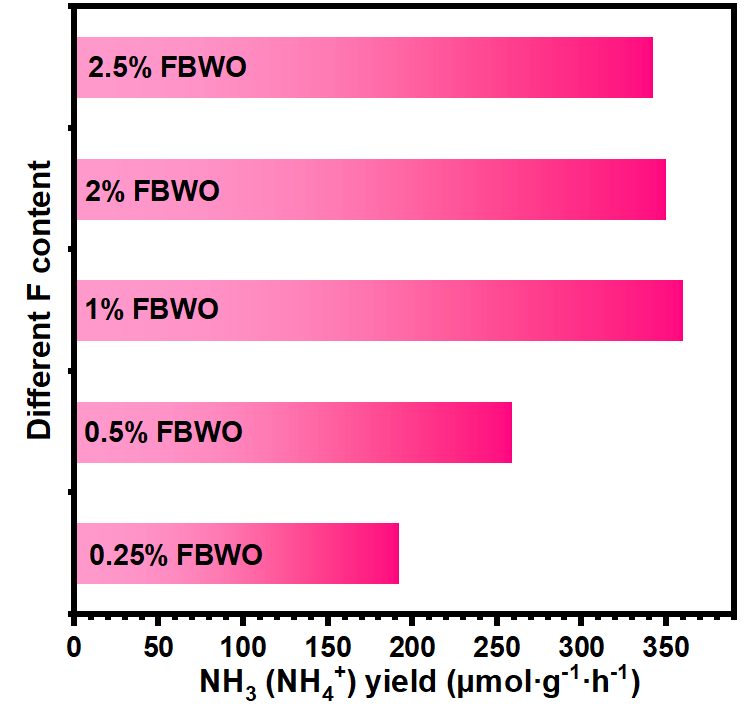


**Figure S22.** NH_3_ evolution over FBWO with varying F contents.


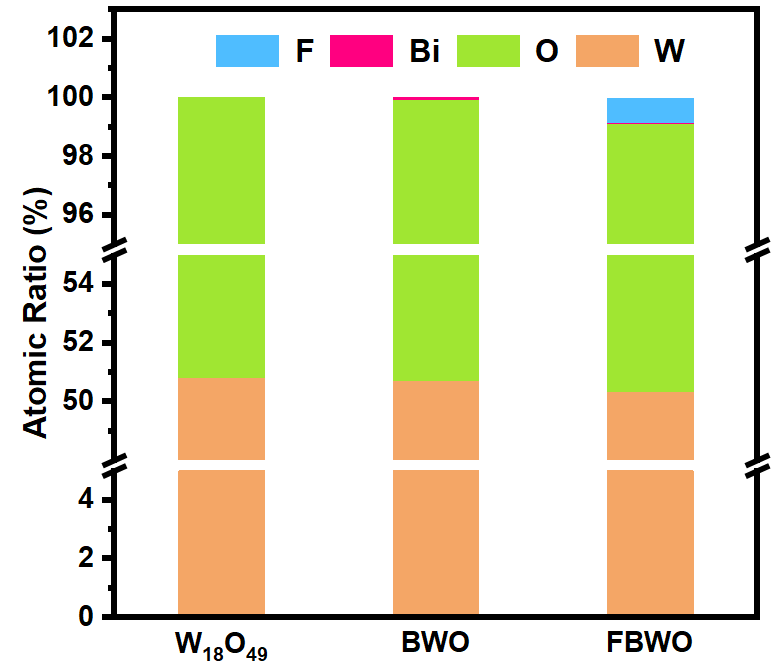


**Figure S23.** Elemental atomic ratios of W_18_O_49_, BWO and FBWO.


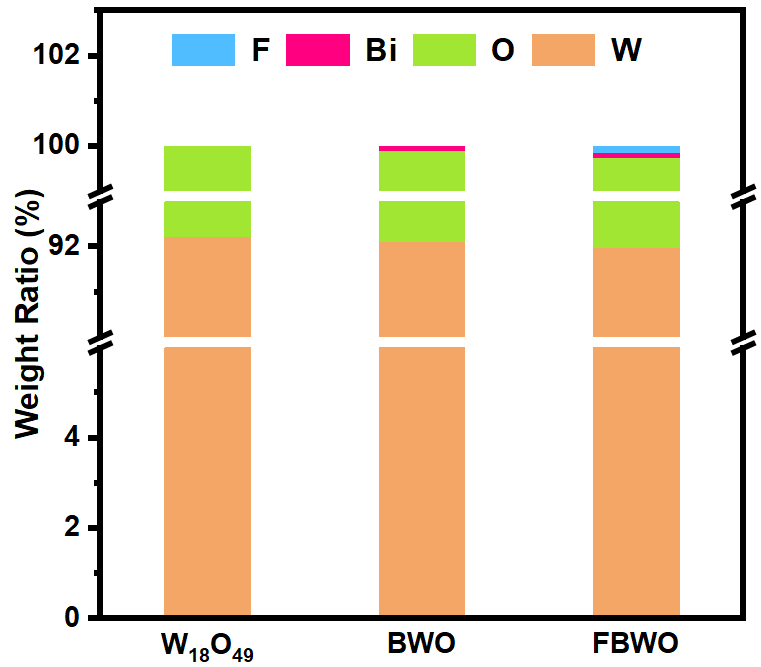


**Figure S24.** Elemental mass ratios in W_18_O_49_, BWO and FBWO.


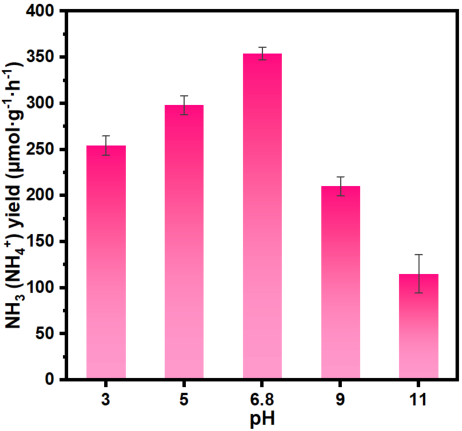


**Figure S25.** NH_3_ evolution over FBWO at different pH values.

**Figure S26.** Schematic of the continuous flow reactor under sunlight or a 300 W Xe lamp light source.

The continuous-flow photocatalytic nitrogen fixation and ammonia production reaction was carried out in a 150 mL quartz reactor. First, 100 mg of FBWO catalyst was added to 1 L of deionized water and ultrasonicated for 1 h to ensure uniform dispersion, forming the pre- reaction solution. The continuous-flow reactor, as shown in the Figure S25, consisted of two peristaltic pumps and a photocatalytic reactor. With a flow rate of 1.6 mL/min from the peristaltic pumps, a circulating solution volume about 100 mL/h was maintained in the photocatalytic reactor. Before the reaction, 80 mL of the pre-reaction solution was introduced into the photocatalytic reactor. Then, high-purity nitrogen gas was bubbled through the system at 150 mL/min for 30 min to remove other gases. Subsequently, the xenon lamp light source was turned on to initiate the photocatalytic reaction, which was recorded every hour.

Over the 200-hour test period, the cumulative nitrogen content in the produced NH_3_ reached 11.41 mg, demonstrating that the nitrogen in the produced NH_3_ originates from the photocatalytic reduction of N_2_ rather than from leaching of nitrogen-containing species from the catalyst itself.

**Table S1.** EXAFS fitting parameters at the Bi *K*-edge various samples (S_0_^2^=0.83)

| samples | path | C. N.^[a]^ | R (Å) ^[b]^ | σ^2^ (× 10^−3^ Å^2^) ^[c]^ | ΔE (eV) ^[d]^ | R factor^[e]^ |
| --- | --- | --- | --- | --- | --- | --- |
| BWO | Bi-O | 2.3±0.6 | 2.05±0.02 | 9.6±2.0 | -1.5±2.3 | 0.02 |
|  | Bi-O | 1.9±0.4 | 2.23±0.02 | 7.8±3.4 |  |  |
| FBWO | Bi-O/F | 2.8±0.7 | 2.06±0.02 | 7.7±3.8 | -2.6±5.7 | 0.02 |
|  | Bi-O | 1.7±0.6 | 2.25±0.02 | 6.3±1.5 |  |  |

^a^C. N.: coordination numbers; ^b^*R*: bond distance; ^c^σ^2^: Debye-Waller factors; ^d^ΔE_0_: the inner potential correction. ^e^*R* factor: goodness of fit.

**Table S2.** The detailed parameters of Figure 4f.

| **Catalysis** | **Feedstock** | | **Reaction temperature** | | **Light source** | | **Reaction solution concentration**  **（g/L)** | | **NH_3_ evlotion rate (μmol g_cat_ ^−1^ h ^−1^)** | | **NH₃ quantification method** | | **Reference** |
| --- | --- | --- | --- | --- | --- | --- | --- | --- | --- | --- | --- | --- | --- |
| **FBWO** | H_2_O, N_2_ | | 25℃ | | > 400 nm | | 0.1 | | 354.21 | | Nessler’s reagent colorimetric | | This work |
| **OVs-BWO** | H_2_O, N_2_ | | room temperature | | > 420 nm | | 1 | | 28.55 | | Nessler’s reagent colorimetric | | 10.1016/j.cej.2021.128827 |
| **Fe-BiOBr** | H_2_O, N_2_ | | 25℃ | | > 400 nm | | 0.5 | | 382.68 | | Nessler’s reagent colorimetric | | 10.1021/acs.chemmater.9b04448 |
| **BiOBr** | H_2_O, N_2_ | | 25℃ | | > 420 nm | | 0.5 | | 104.2 | | Nessler’s reagent colorimetric | | 10.1021/jacs.5b03105 |
| **Fe/W_18_O_49_** | H_2_O, N_2_ | | 25℃ | | > 420 nm | | 0.1 | | 109.9 | | ion chromatography | | 10.1002/anie.202204271 |
| **SiW_9_Co_3_/PDA/Bi_2_WO_6_** | H_2_O, N_2_ | | room temperature | | > 420 nm | | 1 | | 12.06 | | Nessler’s reagent colorimetric | | 10.1039/c9ta13902h |
| **Fe- Bi_2_MoO_6_** | H_2_O, N_2_ | | 25℃ | | > 400 nm | | 0.5 | | 106.5 | | Nessler’s reagent colorimetric | | 10.1016/j.apcatb.2019.117781 |
| **Bi_2_MoO_6_/OV-BiOBr** | H_2_O, N_2_ | | 25℃ | | > 420 nm | | 0.5 | | 81 | | Nessler’s reagent colorimetric | | 10.1039/c9nr02279a |
| **Ag/δ-Bi_2_O_3_** | H_2_O, N_2_ | | 25℃ | | > 420 nm | | 1 | | 1.7 | | Nessler’s reagent colorimetric | | 10.1016/j.jcis.2018.08.091 |
| **AgCl/δ-Bi_2_O_3_** | H_2_O, N_2_ | | 25℃ | | > 420 nm | | 1 | | 202 | | Nessler’s reagent colorimetric | | 10.1016/j.jcat.2019.01.002 |
| **V_O_-BiOBr** | H_2_O, N_2_ | | 25℃ | | > 420 nm | | 0.5 | | 49.04 | | Nessler’s reagent colorimetric | | 10.1021/acs.nanolett.8b03655 |
| **Bi_2_WO_6_/c-PAN** | | H_2_O, N_2_ | | 20℃ | | > 400 nm | | 0.5 | 140 | Nessler’s reagent colorimetric | | 10.1021/acssuschemeng.8b02236 | |

**Reference**

1 Kresse, G. & Furthmüller, J. Efficient iterative schemes for ab initio total-energy calculations using a plane-wave basis set. Physical Review B 54, 11169-11186, doi:10.1103/PhysRevB.54.11169 (1996).

2 Perdew, J. P., Burke, K. & Ernzerhof, M. Generalized Gradient Approximation Made Simple. Physical Review Letters 77, 3865-3868, doi:10.1103/PhysRevLett.77.3865 (1996).

3 Blöchl, P. E. Projector augmented-wave method. Physical Review B 50, 17953-17979, doi:10.1103/PhysRevB.50.17953 (1994).

4 Grimme, S., Antony, J., Ehrlich, S. & Krieg, H. A consistent and accurate ab initio parametrization of density functional dispersion correction (DFT-D) for the 94 elements H-Pu. J Chem Phys 132, 154104, doi:10.1063/1.3382344 (2010).
